# Biomarkers: paving stones on the road towards the personalized precision medicine for oral squamous cell carcinoma

**DOI:** 10.1186/s12885-018-4806-7

**Published:** 2018-09-21

**Authors:** Liang Zhong, Yutong Liu, Kai Wang, Zhijing He, Zhaojian Gong, Zhili Zhao, Yaocheng Yang, Xiaofei Gao, Fangjie Li, Hanjiang Wu, Sheng Zhang, Lin Chen

**Affiliations:** 1 Department of Oral and Maxillofacial Surgery, The Second Xiangya Hospital, Central South University, Changsha, 410011 People’s Republic of China; 2 Department of Dermatology, Hunan Key Laboratory of Medical Epigenomics, The Second Xiangya Hospital, Central South University, Changsha, 410011 People’s Republic of China

**Keywords:** Oral squamous cell carcinoma, Personalized precision medicine, Biomarker, Genomics, Transcriptomics, Proteomics, Epigenomics, Heterogeneity, Microenvironment

## Abstract

Traditional therapeutics have encountered a bottleneck caused by diagnosis delay and subjective and unreliable assessment. Biomarkers can overcome this bottleneck and guide us toward personalized precision medicine for oral squamous cell carcinoma. To achieve this, it is important to efficiently and accurately screen out specific biomarkers from among the huge number of molecules. Progress in omics-based high-throughput technology has laid a solid foundation for biomarker discovery. With credible and systemic biomarker models, more precise and personalized diagnosis and assessment would be achieved and patients would be more likely to be cured and have a higher quality of life. However, this is not straightforward owing to the complexity of molecules involved in tumorigenesis. In this context, there is a need to focus on tumor heterogeneity and homogeneity, which are discussed in detail. In this review, we aim to provide an understanding of biomarker discovery and application for precision medicine of oral squamous cell carcinoma, and have a strong belief that biomarker will pave the road toward future precision medicine.

## Background

In the last few decades, painstaking efforts have been made to fight oral squamous cell carcinoma (OSCC). Medical equipment has become increasingly sophisticated, and our therapeutic approaches have become more standardized and diversified. Despite these advancements, however, disease outcome remains poor, and 5-year overall survival for OSCC is stagnant at 50% [1]. This has prompted us to wonder whether there is something wrong with our diagnosis and treatment. Diagnostic delay for various reasons has resulted in early-stage OSCC patients progressing to an advanced stage [2]. The lack of flexibility in the therapeutic strategy has led to patients suffering from inadequate or excessive treatment [3]. The postoperative follow-up mode of watchful waiting has also deprived most patients with recurrent OSCC of treatment opportunity. We never truly understood our opponent (the tumor), and fought in an ill-advised way.

In fact, it is not difficult to see that OSCC patients have different clinical signs and treatment responses. Even targeted therapy, which has led to major advances for treating tumors, benefits only a subset of tumor patients [4]. Thus, patient heterogeneity provides a major obstacle to correct diagnosis and treatment. To address the heterogeneity of disease, the concept of precision medicine emerged. In 2011, the United States National Academy of Sciences (NAS) presented and systematically discussed the concept of precision medicine and a new classification of diseases based on molecular pathology in a report entitled “Toward precision medicine” [5] . In addition, in the 2015 State of the Union address, President Obama launched the “Precision Medicine Initiative”, further emphasizing that precision medicine would be highly effective for individualized diagnosis and targeted treatment strategies based on individual differences. Biomarkers, which clarify pathophysiological characteristics and reflect individual heterogeneity, can thus undoubtedly serve as paving stones on the path toward precision medicine.

With stimulation by a variety of pathogenic factors, the gene expression pattern of oral mucosal cells changes, and dysfunction of their expression products arises, which accumulate at different stages of cancer progression, leading to the imbalance of gene regulatory networks and eventually inducing malignant transformation [6]. In these seemingly identical malignant transformation processes, different combinations of molecular events give rise to many different clones, which complicate molecular pathogenesis and clinical phenotype considerably. Fortunately, their association with specific molecular events resulted in those tumor clones also having their own distinguishing features [7]. It is therefore expected that these specific molecules, similar to ID cards, will allow us to accurately identify a particular tumor. Biomarkers are what we call “ID cards”. Therefore, an ideal biomarker for use in this context should have the following hallmarks: 1) It can provide an effective diagnosis because its wide occurrence in different histopathological subtypes, clones and stages of a cancer, or because of its specific occurrence in a specific subtype, clone or stage. 2) It can be used to accurately judge the biological behavior of cancer to provide a personalized therapeutic regimen, to estimate the effect of therapy in real time, or to rationally assess prognosis owing to its playing a pivotal role in the development and expansion of tumors and being a so-called “driver” molecule to induce phenotypic alteration of tumor.

## Panning for gold

**Fig. 1:**
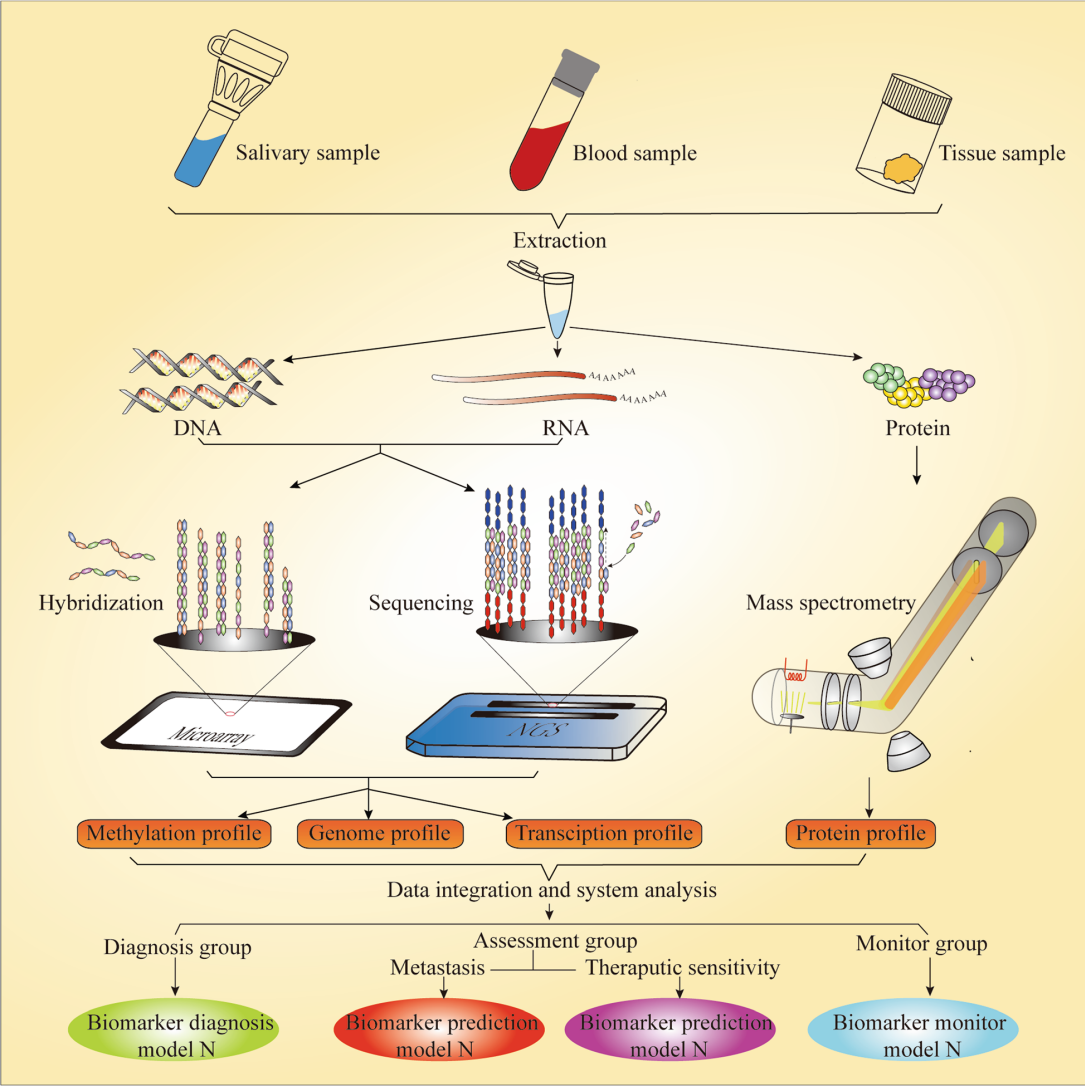
Biomarker discovery with high-throughput technology. The phase of biomarker discovery is fundamental stage for biomarker study in which the landscape of molecular alterations in samples from various sources should be screened. Proteins and nucleic acids such as DNA and RNA are extracted from those samples, which contain almost all molecular information closely associated with cancer. Microarrays and NGS are the most important high-throughput approaches for testing nucleic acids. MS plays a key role in the high-throughput detection of proteins. Comprehensive information including on methylation, as well as genome, transcription, and protein profiles, could be output by these methods. Finally, biomarker models for different applications have been established and classified through data integration and systemic bioinformation analyses. However, strict and large-scale validation testing of these biomarker models is still awaited

Biomarker discovery is a fundamental phase for biomarker study, which is based on comprehensive analysis of the broader landscape of molecular pathological data and clinical pathological parameters. Only deep and widespread mining of data on multiple “omics” levels by various research techniques can lead to the acquisition of a comprehensive set of desired biomarkers for precision medicine and illuminate the pathogenesis of OSCC. The term **“**omics”, a holistic embodiment of the transmission of genetic information, refers to genomics, transcriptomics, proteomics, and epigenomics, encompassing countless molecular events. Therefore, an essential issue is which tool we should use to begin the task of mining gold (biomarkers) from the sand and gravel on a large scale. The rapid development of high-throughput techniques in recent years has provided an approach by which to achieve this (Fig. 1).

### Biomarkers in genomics

The fact that genetic material is continuously replicated means that DNA replication error inevitably occur. Moreover, exposure to factors in the internal or external environment can lead to a range of types of damage to double-stranded DNA. Fortunately, cells are also equipped with an effective DNA repair system and cell cycle checkpoint to monitor and repair damage to DNA molecules. There are indispensable factors for maintaining chromosomal stability. Unfortunately, sustained chronic injury or congenital defects, especially genes related to DNA repair and cell cycle check points, break the balance between damage and repair and contribute to structural or numerical changes in DNA molecules such as point mutations, insertions, deletions, rearrangements, DNA double-stranded breaks gene amplification and aneuploidy, namely chromosomal instability [8–10].

Microsatellites comprise 2 to 6-bp short tandem repeat sequences of highly polymorphic CA dinucleotide, which are located throughout the genome. Replication error and dysfunction of mismatch repair would result in a change in the DNA sequence of microsatellites, which is a special type of gene mutation known as microsatellite instability (MSI) [11]. The frequency of MSI differs among different chromosomal regions, ranging from 10 to 60% in OSCC patients [12]. MSI has been identified as an inactivation mechanism of some of the tumor suppressor genes (TSGs) by causing the accumulation of frameshift mutations in the protein-coding sequence of those TSGs [11]. Interestingly, MSI can also affect the expression of TSGs even if it does not occur in their coding region or promoter [12]. Furthermore, clinical studies in variety of tumors have reported that MSI is linked to a clear clinicopathological profile and has prognostic implications [11]. Unfortunately, there is no large-scale genome-wide study of MSI in the field of OSCC research to date, which is likely one of the reasons that for the lack of ideal predictive markers for OSCC.

Loss of heterozygosity (LOH) is another TSG inactivation mechanism by which the loss of the corresponding wild-type allele of one chromosome turns a heterozygous somatic cell into a hemizygous one [13]. The occurrence of LOH is more common than MSI, with a frequency in some regions in OSCC of up to 80%, which can be detected on chromosome arms 3p, 4q, 7q, 8q, 9p, 11q, 13q and 17p [14]. These regions contain a large number of TSGs, such as TGFBR2 and CNTN4 on Chr. 3p and KDM4C, IL33, PTPRD, SH3GL2, FREM1 on Chr. 9p, or neighboring TSG regions such as the p16/CDKN2 gene cluster, TUSC1 and DMRT2 [14].

The oral and maxillofacial region is provided with an abundant blood supply and has an intricate muscular system. As a result, OSCC has many opportunities to release its genetic material into the blood, whether in the form of tumor cells or microvesicles, under the physical stimulation of external pressure and the squeezing associate with muscle movement. Because such circulating tumor DNA (ctDNA) retains the genetic features of the tumor, the detection of ctDNA could act as a “noninvasive liquid biopsy”. In a study to detect ctDNA in 640 patients with various tumor types, it was found to be detectable in > 75% of patients including those with head and neck cancer [15]. ctDNAs could also be promising as biomarkers to diagnose and monitor OSCC patients.

Saliva, secreted by major and minor salivary glands, is specific body fluid of the oral cavity, which contain various components such as cellular debris, microorganisms, inorganic and organics species [16]. OSCC can secrete tumor-derived DNA into saliva though exosomes or microvesicles [17]. Salivary samples of OSCC patients could also be used to detect these mutated DNA fragments, providing another type of “noninvasive liquid biopsy.” This is particularly promising as researchers have suggested that DNA segments are more easily detected in saliva than in serum or plasma [18].

To discover biomarkers in genomics, it is important to determine which tool would be suitable for the effective and accurate detection of mutated DNA from a large number of genes of OSCC patients. The Cancer Genome Atlas (TCGA) project was launched soon after the Human Genome Project, which opened era of high-throughput genomics analysis. In 2007, genomics research progressed to the stage of global collaboration and International Cancer Genome Consortium (ICGC) was established, which was extremely beneficial for efficiently integrating technology and talents and sharing data [19].

OSCC research has made great strides toward the age of genomics by utilizing high-throughput technology. Next generation sequencing (NGS) has been widely employed by OSCC researchers, because of its advantages in detecting unknown disease-specific mutation sites compared with microarray. NGS is also able to achieve massively parallel DNA sequencing and simultaneous determination of multiple genetic aberrations through sequence alignment and data analysis. Based on available scope of strategy for DNA sequencing, NGS contains targeted gene panel, whole exome sequencing (WES) and whole genome sequencing (WGS). Target gene panel take a variety of enrichment approaches to capture the region of interest to test for genetic mutation. WGS particularly suitable for further analysis to targeted regions that have been preliminarily screened by WGS or WES, on account of the high depth of coverage, the simplicity of subsequent data analyses and cost efficiency [20].Exons contain all the sequence information of proteins, so the application of WES can obtain much of the information related to the individual phenotype. WES can also greatly improve the efficiency of research because only 1% of the whole genome consists of protein-coding sequence. In a recent study, WES was performed on OSCC biopsy samples from users of Arabian snuff, which revealed six novel gene mutation [21]. In another remarkable study by the ICGC, an India team adopted a strategy of combining WES with ultra-deep target sequencing (UDS), which took advantage of the breadth of coverage of WES and the depth of coverage of target sequencing. Several novel gene events were eventually discovered including five specific to OSCC [22]. WGS is able to cover the whole genome sequence which includes not only protein-coding regions but also non-coding regions such as enhancers, promoters, flanking regions and intergenic regions. This make it an optimal strategy for accomplishing comprehensive analysis of the whole genome and the most powerful genomics tool to mine specific biomarkers of OSCC. Unfortunately, WGS has hardly been applied to OSCC biomarker research owing to high cost and labor-intensive of data analysis [23, 24].

### Biomarkers in transcriptomics

There are only 20,000–25,000 protein-coding genes in humans [25]. Under the control of intricate extracellular or intracellular signal and regulatory mechanism, the expression and silencing of these genes can be well organized. Besides, transcripts transcribed from those genes typically undergo a number of processing events such as alternative splicing (AS) before being used as templates for protein assembly.

AS enables a pre-mRNA to produce various transcripts and more than 90% of human transcripts are alternatively spliced. It is thus also an important mechanism for maintaining protein polymorphism [26, 27].However, AS can be utilized by tumor cells to remove certain exons or retain introns, resulting in aberrant protein isoforms and dysfunction. Nonsense mediated RNA decay (NMD) is another important regulatory mechanism at the post-transcriptional level, which enables the monitoring of transcripts and the rapid elimination of mutated transcripts from normal cells. However, the abnormal growth and metabolic speed in cancer result in significant hypoxia, reactive oxygen species generation, and amino acid deprivation in the tumor cell microenvironment. These environmental stresses would inhibit NMD and promote tumor progression [28]. Clearly, neither alteration of gene expression nor transcript variation caused by both of the two above-mentioned mechanisms could be detected at the genome level. Therefore, it is necessary to focus our attention at another level.

Transcriptomics is a realm of research focusing on the gene expression profile and transcript sequences of different cells or tissues, and identifying their alterations. Meanwhile, the physiological and pathological state of cells can be characterized by transcriptomic analysis [29]. Transcriptomic research on biomarkers, just similar to genomics, also requires a high-throughput technique to screen the whole transcriptome of patients to find cancer-specific mutations or genes with altered expression.

Next-generation RNA sequencing (RNA-seq) is an outstanding methodology for this purpose. This technology can not only directly obtain known and novel RNA sequence information from the samples with high-throughput sequencing, with up to 600 Gb generated in a single run, but also quantitatively describe the entire transcriptome [30, 31]. Zhang et al. successfully screened more than 70,000 tumor-related mutations in open reading frames (ORFs) of transcripts and identified six OSCC-specific mutations (ANKRA2, GTF2H5, STOML1, NUP37, PPP1R26, and TAF1L) [32]. Moreover, RNA-seq revealed that TGFBR2 had five alternatively spliced defective forms in OSCC, by which cancer cells evade the anti-tumor effect regulated by the TGF-β signaling pathway [33]. RNA-seq has also been employed by several research teams to accomplish whole-transcriptome sequencing of OSCC and potentially malignant oral disorders (PMDs) or noncancerous matched tissue (NCMT) to establish differential expression profiles between them [34–36].

Microarray, a hybridization-based technology, has been more widely applied to the testing of known transcripts. Its advantages, such as its lower bias and cost, affordable workload of data analysis, and optimized framework of quality control, make it competitive for the quantitative analysis of transcripts, although it has some limitations compared with RNA-seq [37]. Currently, numerous researchers have not only established gene-wide expression profiles of OSCC by utilizing microarrays, but also uploaded their data to public databases such as GEO and ArrayExpress [38–40]. These databases provide free data sharing and even online data analysis, as in the case of Oncomine, which is extremely useful for the mining of biomarkers of OSCC.

### Biomarkers in proteomics

Proteins are the most important macromolecules, given that they directly perform biological functions and are indispensable for orchestrating a cell’s activities and act as dynamic indicators of a cell’s state. Each amino acid is brought in an orderly manner into the ribosome by tRNA, according to a particular codon in the mRNA, and added to a peptide with an ordered sequence. These peptides are folded and assembled into specific structures to give protein a specific biological function. However, translation can be halted or activated, and a protein can be refolded and even degraded under constant pathological stimulation, which we cannot predict just using the gene or transcript sequence. Moreover, a large-scale analysis of tens of thousands of protein events is required to discover biomarkers. Therefore, high-throughput technologies has been rapidly developed for proteomics research.

The primary goal here is to separate proteins from each other before identifying or quantifying them. Two-dimensional gel electrophoresis (2D-PAGE) is a classical tool for this purpose, but it does not meet the requirements of proteomics owing to deficiencies such as the large amount of protein required, the low sensitivity to low-abundance proteins, and the poor separating ability at extreme isoelectric points and molecular masses. Liquid chromatography (LC) not only overcomes the defects of 2D-PAGE but can also be directly coupled with mass spectrometry (MS) to improve the throughput [41, 42]. MS is a current mainstream technology that is widely applied to complete the work of identifying the separated proteins. Numerous strategies for MS are available owing to various ionization methods and mass analysis instruments. It is important to note that electrospray ionization, matrix-assisted laser desorption ionization (MALDI), and surface-enhanced laser desorption ionization are more suitable for protein or peptide samples [43, 44]. Besides, tandem MS is increasingly used for more precise and sensitive mining of biomarkers. MS can now simultaneously perform quantitative analysis based on an isotype labeling strategy such as ^18^O and iTRAQ, while conducting qualitative analysis [44]. In particular, iTRAQ provides much more expansive labeling coverage and can simultaneously accomplish quantitative analysis of eight samples [45].

Proteins are vital building blocks of life, and proteomics has been viewed by a large number of investigators as a promising sphere to discover specific biomarkers of OSCC. Chi et al. screened out one thousand differentially expressed proteins from biopsy samples and completed quantitative analysis of 977 among these proteins with a strategy of ^18^O labeling-based LC-MS/MS [46]. Abé et al. observed that OSCC cells forming the interface between OSCC and normal or dysplastic epithelia (ND) are at a disadvantage in cell “competition” with ND cells and undergo cell necrosis by an unknown mechanism. Laser capture microdissection (LCM) was thus performed to isolate four subgroups of cells adjacent to or far from the interface in each specimen and to screen out a set of specific proteins of the subgroups of cancer cells adjacent to the interface by LC-MS/MS. Finally, it was deduced that these interface-specific proteins are involved in this “competition”. Moreover, the emergence of these proteins in cancer cells would provide a set of biomarkers to announce the “victory” in this “competition” and means that cancer cells confer the ability to metastasize [47]. Recently, the field of spatial proteomics has emerged, in which various techniques such as MALDI imaging mass spectrometry (IMS) are used; this can not only achieve protein identification simultaneously, without utilizing artificial antibodies and labeling, but also present actual spatial abundance of proteins via high-resolution images of tissue slice [48, 49]. Yuan et al. used MALDI-IMS to identify that LRP6 was upregulated in OSCC tissues and could be a promising biomarker for OSCC patients [50].

Blood is the most important medium of the circulatory system and carries a wide variety of substances, which are secreted from every part of the body, including proteins and peptides. Several classic specific tumor proteins in the blood have been used as biomarkers for clinical diagnosis, such as AFP, PSA, CEA, and CA-125. However, to date, no proteins have been identified as specific OSCC biomarkers. One factor that could explain this is that protein abundance in serum/plasma exhibits a wide range of about 10–12 orders of magnitude. Highly abundant proteins such as albumins, immunoglobulins, and haptoglobin and their isoforms and fragments could seriously hinder MS analysis to detect low-abundance proteins such as tumor-secreted proteins at the level of pg/ml. Even if those high-abundancy proteins could be eliminated by technologies such as immunoaffinity depletion, the low-abundance proteins would also be removed nonspecifically [51, 52]. Moreover, oral cancer always induces acute inflammation and the release of a large number of inflammatory factors into the blood, which also hampers the discovery of biomarkers [45]. Despite the challenge of mining biomarkers of OSCC in the blood, researchers are still pursuing this, but much work remains to find circulating biomarkers of OSCC and put them into clinical use [53, 54].

Saliva contains a lower concentration of protein than blood, varying from 1.5 to 2 mg/ml. The concentrations of salivary proteins as biomarkers for OSCC would also be much lower, at the pg/ml level. However, in this context and in contrast to blood, there are no highly abundant proteins that hamper the detection of low-abundance proteins. It is also no longer a problem to test those low-abundance proteins because of the development of isolation technologies and MS [55]. Salivary protein collection is also preferable owing to the constant availability of saliva, the ability to use this method without special equipment or expertise, and increased storage, compared with the collection of proteins from the blood [56, 57]. The study of OSCC biomarkers via proteomic approaches has yielded some progress. In 2008, Wong’s team screened differentially expressed proteins between whole saliva of an OSCC patient and a matched healthy subject using LC-MC and identified them by immunoassays. As a result, they collected sets of salivary biomarkers (CD59, M2BP, MRP14, catalase, and profilin) for the highly effective diagnosis of OSCC [58]. Other salivary biomarkers such as transferrin, myosin, actin, and truncated cystatin SA-I were subsequently discovered and preliminarily identified by different teams utilizing MS and immunochemical technologies [59–61].

### Biomarkers in epigenomics

Epigenetics, a term coined by Conrad H. Waddington, was initially defined as a stable phenotype change without alteration of gene. Through constant innovation of technology and deepening of our understanding of this concept, its definition has been developed and improved to “a stable and inheritable gene expression change regulated by other mechanisms rather than DNA sequence mutation” [62]. Epigenetics has also branched out into epigenomics including DNA methylation, histone modifications, and various non-coding RNAs [63, 64].

DNA methylation is the most widely and intensively studied type of epigenetic change. It not only regulates the differentiation of cells but also plays a key role in the development of tumors [65]. CpG dinucleotides are enriched in genetic promoters and the first exon of genes to form CpG islands (CGIs). However, some CpG dinucleotides are sporadically distributed in the flanking sequences (about 2 to 4 kb) of CGIs to form CpG island shores or CpG island shelves [66, 67]. Cytosines of the CpGs, shores, or shelves can be modified by methylation or demethylation (5hmc) owing to chronic harmful stimulation, such as that resulting from smoking or betel quid chewing. Such modified CpG sites work in association with histone modification to remodel the chromatin spatial conformation, inactivate TSGs, or activate an oncogene [68]. According to Knudson’s two-hit hypothesis, DNA methylation constitutes a second hit to TSGs following gene alteration (mutation, MSI, LOH). DNA methylation, however, is observed at a higher frequency than gene alteration, which is even the only mechanism to inactivate TSGs [69]. An early study revealed that the tally of human CGIs and shores is more than 28 million, and they are widely distributed, covering 40% to 60% of the promoters of oncogenes and TSGs [64]. Therefore, high-throughput tools would be indispensable to search for biomarkers among such a large number of CGIs and shores.

NGS is also a mainstay of research on the methylome. Differentially methylated regions (DMRs), as epigenetic biomarkers, can be screened out by NGS by comparison between cancer tissues and NCMT or PMDs. Whole-genome bisulfite sequencing (WGBS) is currently the only experimental method of unbiased evaluation of the methylation level in the whole genome. The difference between WGBS and WGS involves bisulfite conversion being performed before sequencing, as methylated cytosine is deaminated to uracil more slowly than the unmethylated type. WGBS not only covers the methylation sites across the genome, both known and unknown sites but also achieves single-base resolution. Moreover, its long read length, approximately 500 bases, enables precise alignment between repetitive sequences and reference sequences [70]. Although WGBS has a range of advantages for discovering biomarkers, it still has some limitations. First and foremost, it cannot distinguish between 5mc and 5hmc, which directly affects correct interpretation of the methylation state. Second, methylation sites, despite their abundance, are just a small part of the whole genome, so the analysis of sequencing results for whole genome is clearly unnecessary. Third, high cost of WGBS is also an issue that should be considered [71]. However, the strategy of enriching methylation sites of the whole genome is effective to solve these problems of WGBS. Methyl Cap-seq and MeDIP-seq not only “avoid” 5hmc but also provide coverage of more than 80% of methylation sites, via enrichment of methylated CpG by methyl CpG binding protein and anti-5-methylcytosine antibodies, respectively. However, adopting an “enrichment” strategy is associated with the risk of missing significant methylation sites [72]. A microarray-based strategy has been proved to have high specificity and sensitivity and to involve straightforward data analysis, which is the best approach to detect known methylation sites [73].

In 2013, in a pioneering study by Towle, a microarray was applied to the analysis of DMR among OSCC, dysplastic, and normal tissues. The results show that both hypermethylation events and hypomethylation were significantly increased in the dysplastic and cancer tissues, especially in OSCC. This suggestes that the dysregulated methylation of a large number of genes is involved in the malignant transformation of mucosa. Notably, three genes (TRHDE, ZNF454, and KCNAB3) with a high frequency (90–100%) of methylation specific to OSCC were identified. However, CGIs shore and shelves were not detected in this study [74]. In two other studies, the methylated state was detected not only in CGIs but also in shores, shelves, and exon 1 of genes using the Infinium HumanMethylation 450 K BeadChip. Both studies showed that hypermethylation events occur intensively in the regions of CGIs and shores. However, hypomethylation events were also found to be distributed in shelves, in addition to CGIs and shores. In addition, more new DMRs specific to OSCC were identified, including five hypermethylated (GPR81, THSD7A, ADPRH, VSX1, and SLC35F1) and three hypomethylated regions (KRT6A, TM4SF19, and TMEM132B), indicating that these genes are worthy of further study as potential biomarkers for OSCC [75, 76]. The source of samples for methylation testing would not be restricted to tissues, and saliva is also an excellent choice, given the noninvasive nature of its collection procedure.

Researchers have found that HOXA9 and NID2 are hypermethylated in both salivary samples and biopsy specimens of OSCC patients, and extremely similar diagnostic efficiencies were achieved by detecting those genes in saliva and tumor tissue [77]. Besides, the study of one research team suggested that detecting the methylated state of ZNF582 and PAX1 from saliva and OSCC tissue could also achieve similar diagnostic efficiency [78, 79]. This indicates that an “invasive” biopsy would be superseded by salivary collection for testing the methylated state of genes.

Surprisingly, non-coding RNAs, a class of RNA that cannot encode proteins, account for almost 98% of genes that serve as templates to synthesize RNA, according to a study under The Encyclopedia of DNA Elements (ENCODE) project. They also serve as either regulators to keep the cell running or dysregulators that cause the malignant transformation of the cell [80].

MicroRNAs (miRNAs), which are approximately 18–22 nucleotides long, are the most thoroughly studied noncoding RNAs in OSCC. Single-stranded miRNA can form an RNA-induced silencing complex (RISC) with Argonaute protein to specifically bind to the 3′ untranslated region (UTR) of target RNA, resulting in the degradation or translational inhibition of target RNA [81]. miRNA can also simultaneously regulate several transcripts, while a transcript can be regulated by several miRNAs, which establishes an intricate cross-talk network contributing to tumorigenesis and progression. Detecting the altered expression of miRNAs can thus clearly provide biomarkers for OSCC. In 2002, only a small quantity of human miRNAs had been identified and collected in miRBase. However, there are as many as 2588 human miRNAs in the database (v21, June 2014), thanks to the utilization of RNA-seq [82]. In the field of OSCC, several research teams have established miRNA expression profiles, which have laid a solid foundation for mining biomarkers of OSCC. In tissue samples, two research teams used different microarray platforms to identify differential expression profiles of miRNAs between cancer and NCMT [83, 84]. Moreover, Li et al. analyzed miRNA expression in the serum of OSCC patients [85]. Notably, Momen-Heravi et al. not only systematically screened salivary samples of different pathological groups to find an appropriate endogenous control, miRNA-191, which pushed down barriers to research on salivary-based miRNA biomarkers owing to a suitable endogenous control previously being lacking but also established a salivary expression profile of miRNAs [86]. It needs to be emphasized that RNA-seq should be utilized in the biomarker discovery of miRNA in OSCC for its capability of mining novel and specific miRNA biomarkers and quantification accuracy.

Recently, long noncoding RNA (lncRNA) has rapidly become a focus of research in the field of cancer because of its powerful regulatory ability, involvement in all “omics” spheres, and larger quantity (172,216 transcripts of lncRNA), in contrast to miRNA. Therefore, detecting altered expression of lncRNAs can more clearly elucidate tumorigenic mechanisms than miRNAs. However, lncRNA research in oral cancer has only reached a preliminary stage [87, 88]. Substantial work remains to be done for biomarker discovery in this promising field.

## New yardstick for diagnosis and treatment assessment

**Fig. 2:**
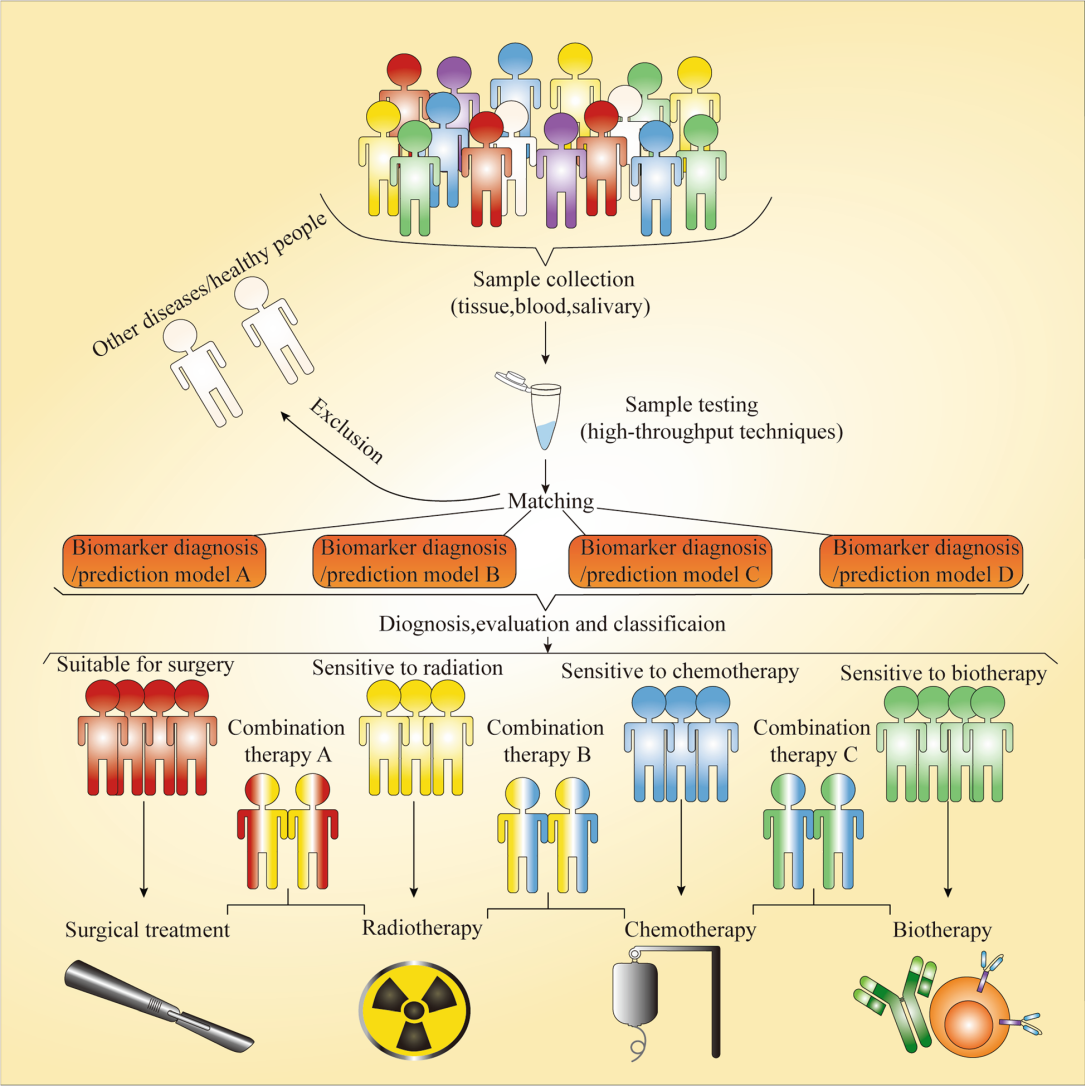
New yardstick for diagnosis and treatment assessment. Samples from individual who consult doctor are detected by various high-throughput techniques to achieve the personalized molecular information. Oral cancer patients will be diagnosed and healthy or other disease patients excluded by matching patient molecular alternation with various diagnosis models. According to the biomarker prediction models, oral cancer patients then could be evaluated and classified into most appropriate group to receive the most reasonable therapeutics

In conventional clinical procedures, the diagnosis of oral OSCC cancer, especially at an early stage, relies on the experience of the dentist and pathologist. Pretreatment assessment requires only imaging and physical examination to roughly judge the cancer stage. Posttreatment monitoring completely depends on the clinical manifestations and imaging modality. However, such diagnostic methods and treatment assessments are so extremely subjective and have low sensitivity that misdiagnoses and unreasonable assessments frequently occur, which inevitably results in poor prognosis of OSCC patients. A biomarker-based diagnosis and prediction model can not only accurately identify the true identity of tissues to assist us with diagnosis and posttreatment monitoring but also reflect the biological behavior of cancer cells to help us choose the most appropriate therapeutic regimen (Fig. 2). In addition, the rapid development of high-throughput techniques has made sample testing more efficient and cost-effective. Accordingly, biomarkers would undoubtedly be the new yardstick for diagnosis and treatment assessment.

### Early diagnosis

It is essential to recognize that early diagnosis is a prerequisite for improving the cure rate and promoting the quality of life of OSCC patients. Patients at an early stage have a better survival rate (approximately 90%) with single surgical therapy and, for them, dysfunction caused by tissue defects can be completely compensated. In contrast, patients at an advanced stage have to undergo multidisciplinary synthetic and sequential therapy, which gives rise to longstanding pain and mental trauma. Moreover, less than half of them survive and they have a poor quality of life [89, 90]. Unfortunately, a delay in diagnosis by up to 6 months is common owing to patients’ lack of awareness of the need to visit a doctor, inaccurate diagnosis by inexperienced dentists and the tedious procedure of various examinations [91, 92].

Molecular events related to tumorigenesis have already occurred before the appearance of visible lesions and typical clinical symptoms. Testing for those early events can facilitate the early screening of OSCC and provide reliable evidence to actively handle such potentially malignant lesions. As early as 2000, LOH on 3p, 9p21, or 17p was identified as an early event that drives the malignant transformation of PMDs [93]. Mao et al. took the lead in using a microarray to test tissues of OSCC and PMDs and developed a 29-transcript prediction model that offers a more precise prediction of the prognosis of PMDs than clinicopathological risk factors [94]. Another research team applied a microarray combined with quantitative real-time reverse transcription-polymerase chain reaction (qRT-PCR) to identify the value of applying ISG15, which continuously increases during tumorigenesis, for early diagnosis [95]. Excellent progress has also been made in salivary quantitative proteomics in the investigation of biomarkers for early diagnosis. For instance, Griffin’s team revealed that myosin and actin are promising saliva biomarkers for the early detection of OSCC [59]. A double-blind multicenter prospective study also unequivocally showed that P16 gene methylation as an initiating event of OSCC is a remarkable predictor of the malignant transformation of PMDs [96]. Notably, research at Johns Hopkins Medical Institutions indicated that detecting hypermethylation of EDNRB and DCC has the same performance as an expert clinical examination for the early diagnosis of OSCC, which indicates that limited expert health care resources need not be an obstacle to the early and precise diagnosis and screening of OSCC [97]. miR-31 has also been identified as an initiating event in OSCC due to being observed in PMD patients with a higher risk of malignant transformation [98, 99]. In addition, miR-31, miR-16, let-7b, miR-338-3p, miR-223, and miR-29a are considered as potential noninvasive biomarkers for early diagnosis of OSCC [100, 101].

### Pretreatment assessment

Personalized therapy is required to achieve precise pretreatment assessment, including assessing the individual and the tumor. The majority of researchers have focused more on studying the state of the tumor, including the tumor’s ability to invade and metastasize and its sensitivity to therapy.

We noted in routine clinical work that some cN0 OSCC patients have extensive lymph node metastases (LNMs) or even extracapsular spread, as reported by postpathological diagnosis. Furthermore, several studies have shown that 20–40% of cN0 OSCC patients have occult metastases [102, 103]. At present, the depth of invasion (DOI) is the most reliable clinical parameter for the prediction of occult nodal metastasis and biopsy is the most feasible approach to measure it. However, data from one study showed that the DOI of OSCC biopsy samples was poorly correlated with the actual DOI of the entire tumor [104]. In addition, sentinel lymph node biopsy (SLNB) is the most straightforward method for detecting occult metastasis. However, its popularization has been difficult because of technological difficulties caused by the complicated anatomical structure of the neck and the lack of a tracer instrument [105]. We should thus shift our version to those molecules that are directly related to invasion and metastasis for precise pretreatment assessment. LOH, as mentioned above, is a high-frequency event on chromosome 13q. Ogawara et al. further mapped this LOH to 13q14.3 and identified a significant relationship between it and LNM [106]. An invasive tumor front (ITF), three to six cell layers, or detached cell groups at the advancing edge of the tumor have been identified as a group of cancer cells with the most invasive and metastatic ability in epithelial tumors [107]. Abnormal expression of SERPINE1 and SMA in ITF indicates not only LNM but also extracapsular spread (ECS). Surprisingly, detecting SERPINE1 (95%) and SMA (82%) showed more sensitivity for the examination of ECS than MRI (7%) [108]. Moreover, a multigene signature for predicting LNM in OSCC was transferred to a gene diagnostic microarray in a large multicenter cohort study. The validity of this diagnostic microarray was verified by a study on a large number of samples of cN0 OSCC patients, achieving high performance with 86% sensitivity and 89% negative predictive value (NPV) for assessing LNM; this is almost on par with the performance of SLNB [109]. Recently, the 22-gene LNM signature identified by another study with a large number of samples was applied to pretreatment evaluation of early-stage OSCC patients and effectively reduced the rate of overtreatment by two-thirds [110].

Inherent or acquired chemoresistance mechanisms lead to chemotherapy failure for some OSCC patients. Moreover, tumor cells possess different levels of chemosensitivity to different chemotherapy agents. For example, most oral cancers are inhibited by cisplatin (CDDP), but few are sensitive to methotrexate. Histoculture of OSCC and chemosensitivity test in vitro are currently the most effective ways to achieve accurate pretreatment assessment. However, histoculture in vitro requires the isolation of cells from tumor tissues, which has some flaws such as cell trauma, contamination, changing the microenvironment, and being time-consuming [111]. The direct detection of biomarkers that reflect the chemosensitivity or chemoresistance of an individual could overcome these flaws of histoculture and provide a timely personalized regimen. For instance, an association of dihydropyrimidine dehydrogenase, the rate-limiting enzyme of 5-Fu catabolism, with the sensitivity to 5-Fu has been observed in 103 patients with OSCC [112]. Moreover, the expression of survivin was shown to predict chemosensitivity to both CDDP and 5-Fu in OSCC [113].

At present, there is no effective clinical variable for evaluating the radiosensitivity of OSCC patients. The implementation of radiotherapy for patients with a tumor enriched in cancer stem cells (CSCs) not only results in a poor treatment effect but also brings numerous side effects because the mechanisms behind the radioresistance of CSC, such as the elimination of reactive oxygen species (ROS), cell cycle regulation, enhancement of DNA damage response, and hypoxic tolerance, facilitate OSCC survival and repopulation [114]. Recently, a 13-gene expression-based radioresistance score model was established by integrated analysis of multiple head and neck squamous cell carcinoma (HNSCC) datasets from TCGA. This valuable strategy should be applied to establish a prediction model for OSCC [115].

By achieving the precise killing of tumor cell with few side effect, biotherapy, an emerging method, has received increasing attention. Programmed death receptor ligand-1 (PD-L1), a notable inhibitor of the activity of T lymphocytes, is widely expressed in many normal tissue cells such as lung, skeletal muscle, and even lymphoid tissues. However, tumor cells emulate normal cells to produce PD-L1 and insert them into their own membrane surface to evade immune surveillance and become more invasive [116, 117]. In recent years, anti-PD-L1 has been proved to provide considerable efficacy for tumor control in patients with PD-L1 overexpression [118, 119]. The obtained data demonstrated that more than two-thirds of OSCC cases overexpress PD-L1. In other words, the majority of OSCC patients would benefit from anti-PD-L1-based biotherapy [120].

### Posttreatment monitor

How best to monitor posttreatment patients is a difficult issue. At present, there is no effective way to detect tumor recurrence at an early stage. This is because inflammatory granulation tissues and flap reconstruction make physical examination difficult, impeding the accurate completion of differential diagnosis. Imaging technology also has numerous limitations, despite having made great progress. For example, contrast-enhanced computed tomography is required for follow-up observation of suspicious change over 6 months and positron emission tomography, a more reliable examination approach, was shown to be unable to distinguish tissue repair and tumor recurrence 3 months after an operation [121, 122]. However, pronounced growth of residual OSCC cells can take place 1 month after an operation [123]. Accordingly, it is not until patients exhibit typical clinical signs that tumor relapse is diagnosed. By that stage, the majority of patients with recurrence have already lost the opportunity to be cured.

Fortunately, some improvements in this situation have emerged. ctDNA as a real-time reflector of tumor burden has been utilized for postoperative monitoring of various tumors and its test sensitivity and specificity are far superior to those of imaging technology [124]. In the field of oral cancer, Hamana et al. detected MSI and LOH on ctDNA of OSCC patients in 2005. They found that ctDNA of some postoperative patients could be detected within 4 weeks after operation and all of those patients died during 1 year in a follow-up study [125].

## Heterogeneity and homogeneity should be considered

OSCC is a fairly complicated tumor, which means not only that tens of thousands of molecular aberrations are involved in its tumorigenesis but also that OSCC patients display significant heterogeneity and homogeneity. As such, numerous factors from sample preparation to clinical application of biomarkers should be of concern for more efficient and accurate discovery and rational application of biomarkers.

### Heterogeneity

#### Tumor heterogeneity

**Fig. 3:**
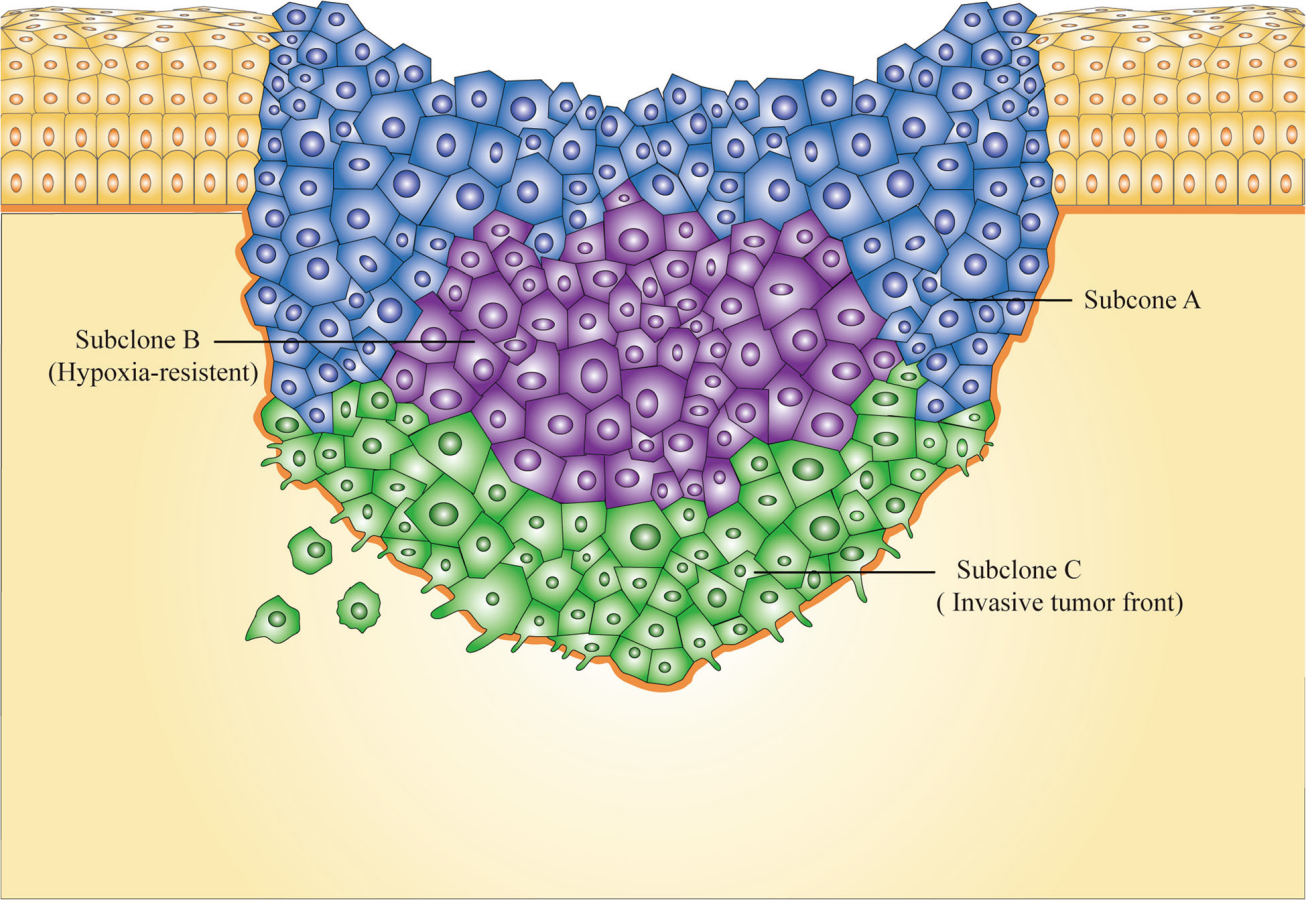
Intratumor heterogeneity. In the context of the stresses from the external environment and the need for nutritional sources, onco-evolution takes place in the solid tumor and various subclones form. Subclone B, for example, is a group of hypoxia-resistant tumor cells and subclone C is another group of invasive tumor cells known as ITF. These subclones undoubtedly possess their own molecular characteristics which poses a formidable hurdle in the process from biomarkers discovery to application

A previous study suggested that heterogeneity was observed in the histological phenotype of OSCC tissue specimens [126]. Then, via an analysis at the molecular level, Bhawal et al. noted that tumor cells with positive expression of 14–3-3σ, a key gene for the G_2_/M checkpoint, constituted only part of the total tumor cells in the OSCC specimen [127]. This is because OSCC, like other epithelial-derived solid tumors, seems to be driven to undergo a complicated process of Darwinian-like evolution by environmental pressures such as hypoxia, acid-base imbalance, and malnutrition. On the one hand, this evolution facilitates the survival of tumor cells in such a difficult living environment. On the other hand, various subclones appearing in a single tumor tissue cause intratumor heterogeneity, which poses a formidable hurdle in the process from biomarker discovery to application (Fig. 3). [128]. Moreover, it is currently unclear how those different OSCC cells are distributed in tumor tissue [129]. However, it is becoming clear that cells in ITF are a subgroup that are clearly distinct from other regional cells in OSCC tissue and can be easily captured by LCM. For example, Wang et al. performed LCM to collect intratumoral cell subgroups from different parts of the tumor (ITF, center, surface) and found that LOH on 9p21 (RPS6) was more frequently detected in ITF than at the center and surface [130]. Notably, brush biopsy is a noninvasive approach for the acquisition of tumor cells and can effectively solve the problem caused by intratumor heterogeneity, which is probably an optimal method for the early diagnosis of OSCC [131].

Squamous cell carcinomas of oral, oropharynx, laryngopharynx, and larynx have always been considered to be HNSCC, without individual analyses of them, in many biomarker studies. However, significant differences in clinical features and therapeutic sensitivity have been noted among these sites [132]. Molecular differences between those locations have also been screened out by several studies at different molecular levels [69, 133, 134]. As such, intersite heterogeneity should clearly be taken into account in the process of sample collection or data analysis. Otherwise, specific biomarkers could easily be missed.

**Fig. 4:**
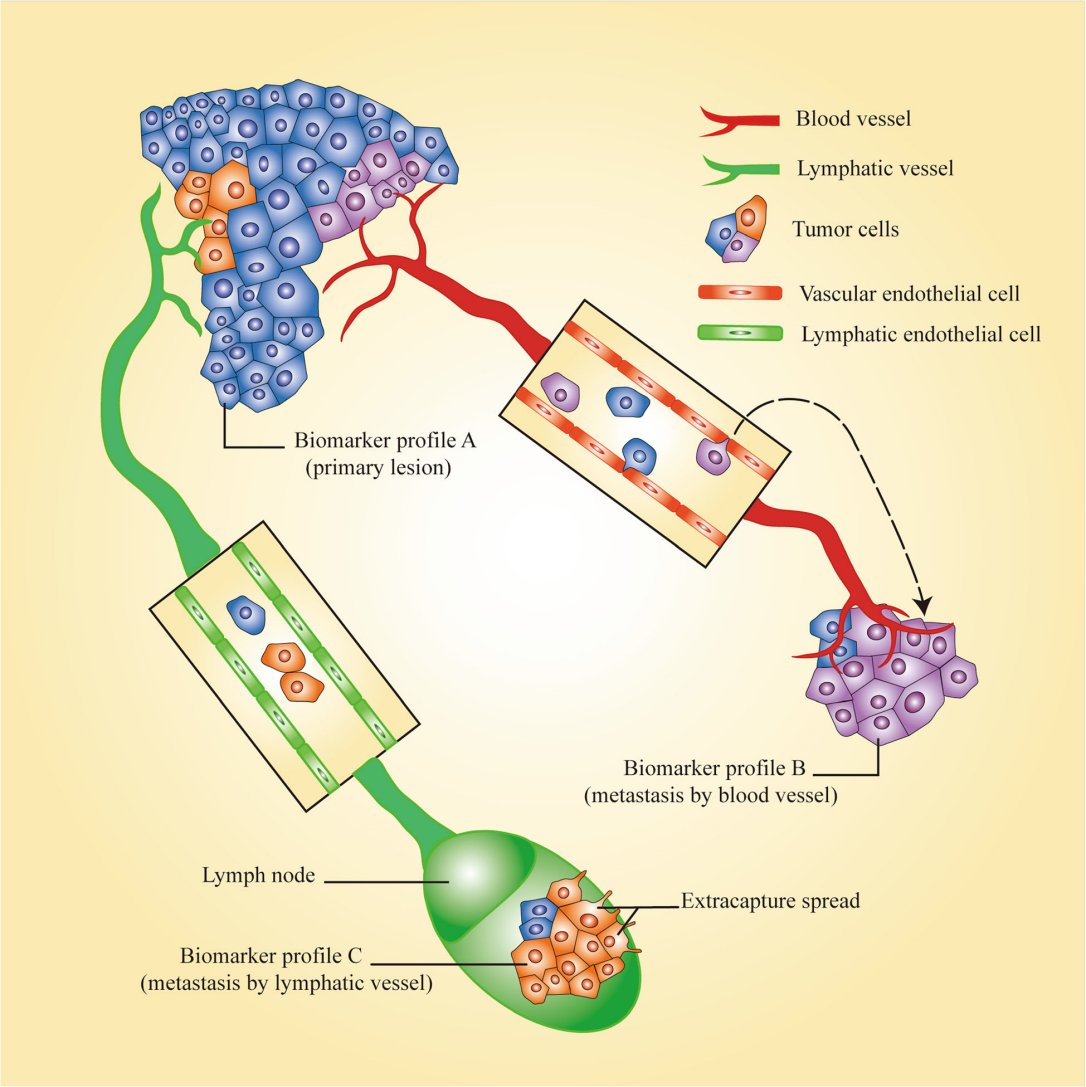
Metastatic heterogeneity. The minor clone in the primary lesion turns into a major clone in metastatic lesions. In this way, biomarkers of primary lesions could not be used to accurately estimate the state of metastatic lesions

Unfortunately, tumor heterogeneity also gives rise to many more problems. Tumor cells have generally been thought to be capable of invasion and metastasis at an advanced stage, but tumor cells have already disseminated at an early stage in some OSCC cases. Moreover, new clones would form in the metastatic lesion, or the major clone in the primary tumor would be replaced by a minor one, according to the model of parallel evolution. A metastatic lesion could thus not be accurately evaluated using a biomarker identified from the primary lesion (Fig. 4) [135]. A research team in Denmark recently undertook UDS to study metastatic lesions and primary lesions of OSCC and to identify mutations; they demonstrated extreme similarity between the two types of lesions. However, the number of samples was far too small and the study was performed only at the genomic level, preventing definitive identification of whether parallel evolution occurs in OSCC patients [129].

**Fig. 5:**
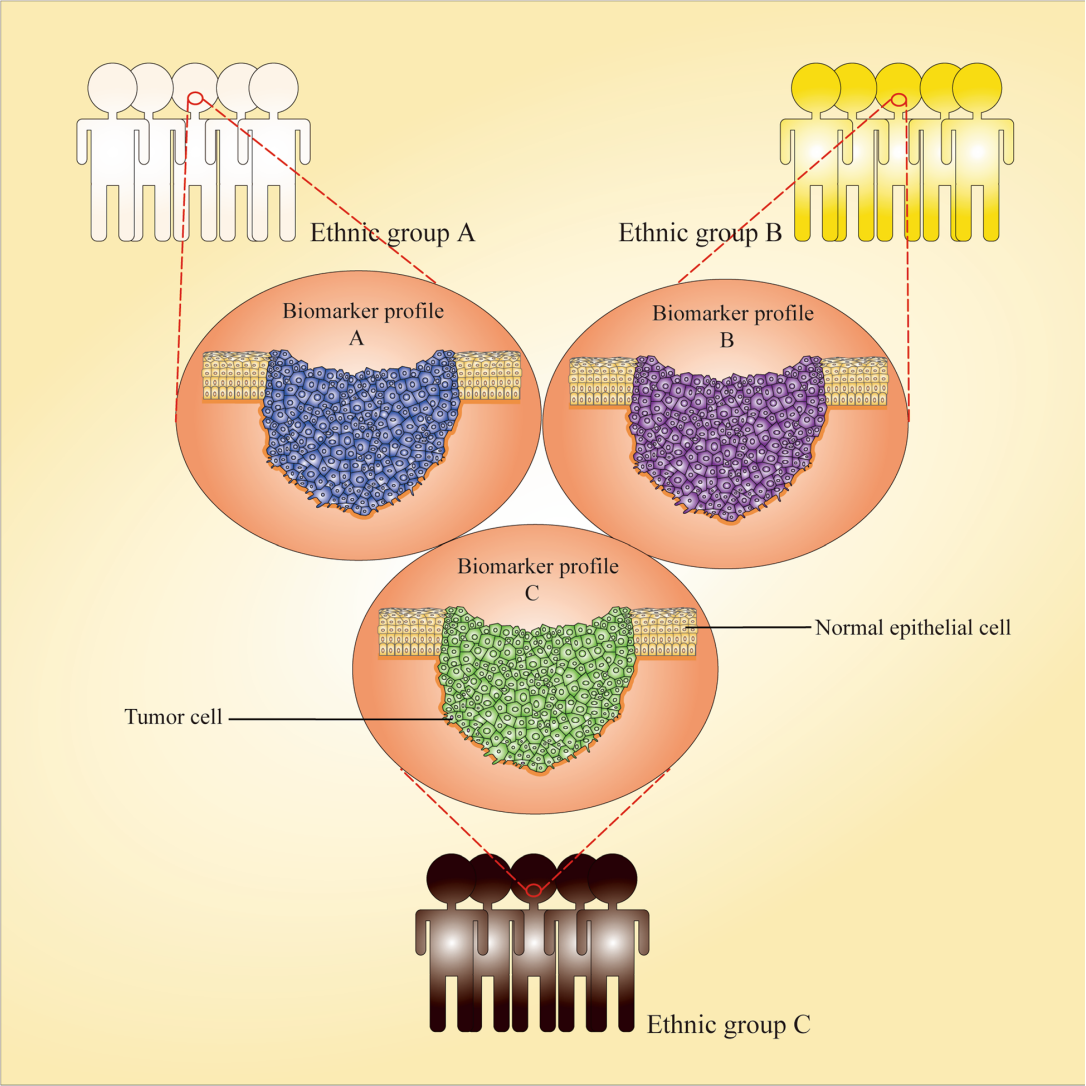
Inter-ethnic heterogeneity**.** A biomarker model that had not been validated in all ethnic groups would not be suitable for other groups owing to the heterogeneity among them

Furthermore, it should be emphasized that heterogeneity between ethnic groups hinders the range of applications of some biomarkers (Fig. 5). Because ethnic groups with the same geographical residence are exposed to similar environments, diets, lifestyles, etc., which result in a high probability of presenting similar “omics” variation in one ethnicity [136, 137]. A gene expression profile study presented significant heterogeneity between UK and Sri Lankan OSCC patients [138]. In a salivary proteomic study, Csösz et al. identified heterogeneity of OSCC protein biomarkers in different ethnic groups [139]. In addition, Basu et al. also identified a set of hypomethylated genes that are specific to OSCC tissues of Indian patients [75]. Thus, we should not only consider the Inter-ethnic heterogeneity when collecting samples or analyzing data for every specific biomarker, but also avoid directly applying a biomarker prediction model established for one ethnic group to others before large-scale sample identification for accurate diagnosis and assessment.

In fact, there is some evidence showing the intra-ethnic heterogeneity caused by human migration in other solid tumors. That is, because the immigrant may undergo the alteration of cues such as the environment that obviously differ from the circumstance that their ethnicity lives [140]. Further study will be necessary for us owing to the lack of evidence of Intra-ethnic heterogeneity in OSCC. Until we come to a conclusion, it should be taken into consideration to develop strict inclusion criteria for the sample especially in studies with small sample sizes such as the establishment of a training set.

Finally, it is important to note that recurrent lesions also demonstrate molecular divergence from the primary lesion owing to clone evolution. For example, further progression with regard to the heterogeneity of OSCC has been reported by the above-mentioned Danish team in a recent study, which specifically suggested a total of 10 of 24 somatic mutations screened out by WES and UDS were specific to recurrent lesions [141]. In other words, some biomarkers applied to primary tumor diagnosis or evaluation may not be suitable for the diagnosis or monitoring of recurrence when a new clone becomes the major subclone in a recurrent lesion. A screening strategy with greater coverage should thus be applied in postoperative monitoring.

Various databases based on omics study have now been established to provide huge amounts of free high-throughput data for different ethnic groups. We can perform comprehensive analyses of those data using bioinformatic and statistical methods to overcome the heterogeneity of pathogenic factors and ethnic groups and efficiently and cost-effectively screen out “homogeneous” biomarkers applied to the diagnosis of “span” OSCC patients without regard for heterogeneity. A meta-analysis of microarray data is the best example of this [142]. Moreover, those biomarkers could be efficiently translated into clinical application owing to attractive application prospect.

Literature-based discovery of biomarkers is another available approach. For example, Sharma et al. presented a comprehensive map of gene alterations closely related to OSCC obtained by a retrospective search of the literature in the PubMed database and statistical analysis [143].

#### Microenvironment heterogeneity

**Fig. 6:**
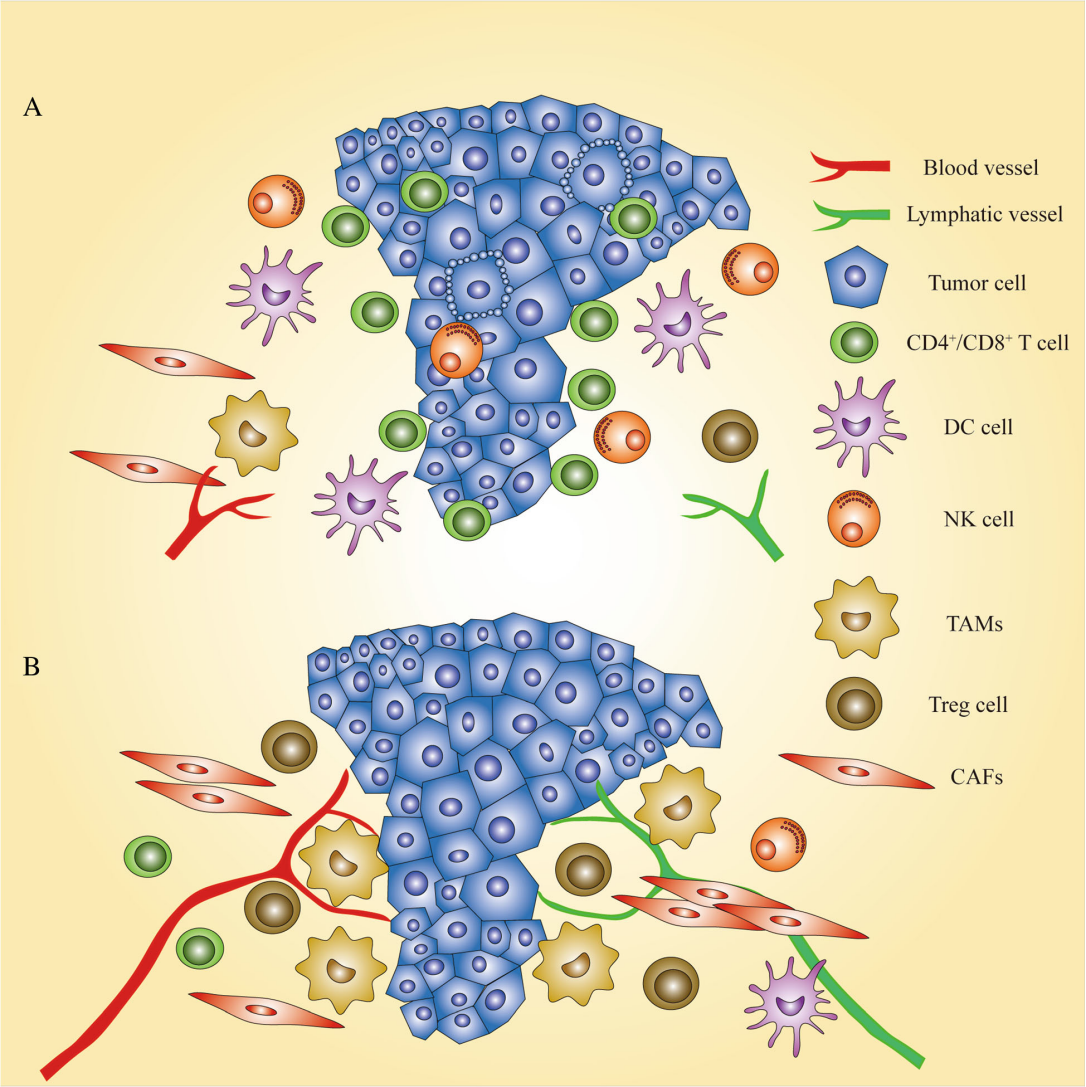
Microenvironmental heterogeneity. Tumor microenvironment mirrors the organism’s response to tumor cells. **a**. When anti-tumor cells such as CD4^+^/CD8^+^ T cells, DC cells, and NK cells infiltrate tumor tissues, tumor cells could be trapped in the local region and even induced to undergo apoptosis. **b** However, when enriched in or surrounding tumor tissues, tumor-associated macrophages (TAMs), Tregs, and CAFs can facilitate tumor growth and metastasis by lymphangiogenesis and hemangiogenesis induced by them. Moreover, CAFs can generate a physical barrier to protect tumor cells from damage caused by drugs and ionizing radiation. As such, it is imperative to mine biomarkers used to evaluate the state of TILs and stromal cells

We have focused more attention on tumor cells during the progress made in biomarker discovery, but have neglected a panel of non-tumor cells associated with tumors. They surround the tumor tissue and interact with tumor cells to construct a hospitable environment for tumor growth and invasion, and can be categorized as tumor-infiltrating lymphocytes (TILs) and stromal cells (Fig. 6) [144].

TILs are an immunocyte family (nature killer cells, T cells) with remarkable heterogeneity and a close association with immune reactions to tumors. In histological research, a low density of infiltrating nature killer cells and the absence of intraepithelial micro-abscess formation have been observed in some advanced OSCC patients and predict a poor prognosis [145, 146]. Basu et al. utilized a microarray to analyze genome-wide DNA methylation and incidentally found that a set of hypomethylated genes are associated with T-lymphocyte regulation inducing anti-tumor immune responses, and higher expression of those genes was associated with a favorable prognosis [75]. T regulatory cells (Tregs), a member of the TIL family, act as inhibitors of anti-tumor response. CD4+ CD25+, Fox3+, and CTLA-4 are important biomarkers for recognizing Tregs. They have been found to be enriched in some patients’ OSCC tissues and related to high-grade malignancy [147].

Stromal cells consist of histocytes such as fibroblasts and antigen-presenting cells. Cancer-associated fibroblasts (CAFs), a known subset of fibroblasts, greatly contribute to tumor progression and metastasis and their activity is significantly related to the prognosis of OSCC patients [148]. The lack of a specific biomarker for CAFs has impeded research progress in this field. However, Kartha et al. successfully screened PDGFRβ as a biomarker for CAFs, which is far superior to traditional biomarkers (podoplanin and αSMA). It also acts as an indicator of an activated state of CAFs, which is an important foundation for further biomarker discovery in CAFs [149]. Recently, PD-L1 was also found on the surface of the CAFs of some patients. Findings have shown that CAFs with PD-L1^+^ status serve as an “accomplice” to assist tumor cells in repressing cytotoxic T-lymphocytes and resulting in poor prognosis for those patients [150].

Finally, heterogeneity of TILs and fibroblasts induces differences of therapeutic sensitivity between cancer patients. For instance, a novel subset of CCR2^+^ Tregs has been defined to be significantly associated with immune escape of OSCC and low-dose cyclophosphamide could eliminate those Tregs and enhance the priming of cytotoxic T lymphocytes. Detecting CCR2 in biopsy samples would act as an indication for chemotherapy [151].

It can be seen that TILs and fibroblasts mirror the organism’s overall “attitude” of facilitating the progress of tumor cells or killing them. It would be too simplistic to evaluate a patient’s disease state just according to the state of tumor cells; this would inevitably lead to patients being over- or undertreated, resulting in difficulty achieving a pronounced therapeutic effect and having a negative impact on the patient’s quality of life. In this context, the integration of multidimensional (omics) biomarkers of tumor cells, TILs, and stromal cells is an appropriate approach to achieve personalized and precise assessment of OSCC patients.

### Homogeneity

#### Clinical-phenotypic homogeneity

In a xenograft mouse model study, the investigators observed that metastatic lesion-derived OSCC cell lines (OSC19 and OSC20) present similar invasion and metastasis abilities, but their expression profile and immunophenotypic properties were markedly diverse [152]. This suggests that inter-patient molecular heterogeneity would be accompanied by clinical-phenotypic homogeneity. In other words, the coverage of one or a set of biomarkers would be extremely limited. However, the systematic classification of these prediction models in terms of the homogeneous clinical phenotype would effectively address this problem.

#### Inter-disease homogeneity

OSCC patients are still dominated by the middle-aged and elderly, although this disease is also rapidly increasing in the young [153]. As a result, most patients who consult a doctor suffer not only from OSCC but also from systemic disease. If molecular events that could act as biomarkers for OSCC diagnosis are simultaneously present in another disease, this would be bound to curb the diagnostic efficiency of the biomarker, especially in body fluid. Such biomarkers are too numerous to mention all of them individually here. However, visfatin is one example, that is upregulated in the blood of both OSCC and chronic kidney disease patients [154, 155]. Moreover, C-reactive protein (CRP), an index of systemic inflammation, is overexpressed in the serum of OSCC and type 2 diabetes mellitus (T2DM) patients [156, 157]. The composition in saliva is similar to that of the blood owing to the exchange of material between them. Therefore, a high level of CRP has also been found in OSCC and T2DM patients [158, 159]. In addition, in some studies, it has been claimed that patients with Sjögren’s syndrome also share the same molecular events in saliva as OSCC patients [160, 161]. To resolve these issues, it would be worth applying the abovementioned strategies of utilizing a public database to discover disease-specific biomarkers. Avoiding the use of “homogeneous” biomarkers that overlap with systemic disease or restricting the range of applied biomarkers is also available as an option to improve the diagnostic performance.

## Conclusion

Personalized precision medicine of OSCC is like a map for a traveler (patient) explaining the path he needs to take. These roads are paved with biomarkers. To create these maps, we as doctors also need to embark on a journey to search for biomarkers. This journey will undoubtedly be a long one owing to the time-consuming and laborious work of biomarker discovery. Fortunately, the availability of high-throughput technology hastens our biomarker discovery from the complex realm of molecular pathology. Compared with semiquantitative parameters provided by pathological diagnosis, biomarkers could supply absolute and precise quantifiable parameters for diagnosis. We can obtain information on the biological characteristics of cancer cells and the immune state of the patients by using biomarkers for comprehensive and personalized assessment. Furthermore, the subjectivity of physical examination and imaging diagnosis could be overcome through the use of objective evidence from biomarkers.

To date, several guidelines for biomarker study have been established, that provide a standard protocol for sample collection and storage, rational study design, detailed methods, suitable analysis strategy etc. Furthermore, with the improvement of technology and algorithms, high-throughput platforms offer data with high accuracy, sensitivity and reproducibility, and platform-dependent differences in molecular measurement have been effectively reduced [162–164]. Nevertheless, the progression of biomarkers in OSCC is still stymied by some factors. First, there are few large-scale studies based on high-throughput technology to examine the “omics” landscapes of OSCC and no public database specific to OSCC offers molecular profiles of various “omics”, which means that the foundation of biomarker study in OSCC is not yet laid. Second, the majority of biomarker studies in OSCC are still the development of single-molecular type biomarkers and few investigators seem to shift their focus to biomarker panels. However, it has been identified that multi-molecular biomarker panels integrating 2 or more molecular information (predictor) into one predictive model significantly improve diagnostic accuracy and enhance the predictive power in various tumors. Furthermore, multi-omics type biomarker panels containing various “omics” molecular events are more attractive due to their presentation of a more genuine state of tumor cells. Third, although many study results indicate that OSCC is a heterogeneous solid tumor, a large number of investigators still develop a biomarker without considering heterogeneity. Moreover, no article elaborately discusses the homogeneity of clinical phenotypes in different molecular profiles and molecules in different diseases.

Accordingly, much need to be done on the path to precision medicine of OSCC. Several suggestions are as follows: establishing the multiple “omics” database based on high-throughput technology and publicly available for researcher; employing integrated analysis of various “omics” or disease data to develop biomarker panels; completing a biomarker study on the premise of realizing the heterogeneity and homogeneity of OSCC; a need for further study of the heterogeneity and homogeneity of OSCC.

## Abbreviations

2D-PAGE: Two-dimensional gel electrophoresis
AS: Alternative splicing
CAFs: Cancer-associated fibroblasts
CDDP: Cisplatin
CGIs: CpG islands
CRP: C-reactive protein
CSCs: Cancer stem cells
ctDNA: Circulating tumor DNA
DMRs: Differentially methylated regions
DOI: Depth of invasion
ECS: Extracapsular spread
ENCODE: Encyclopedia of DNA Elements
HNSCC: Head and neck squamous cell carcinoma
ICGC: International cancer genome consortium
ITF: Invasive tumor front
LC: Liquid chromatography
LCM: Laser capture microdissection
LncRNA: Long noncoding RNA
LNMs: Lymph node metastases
LOH: Loss of heterozygosity
MALDI: Matrix-assisted laser desorption ionization
MS: Mass spectrometry
MSI: Microsatellite instability
NAS: National Academy of Sciences
NCMT: Noncancerous matched tissue
NGS: Next generation sequencing
NMD: Nonsense mediated RNA decay
OSCC: Oral squamous cell carcinoma
PD-L1: Programmed death receptor ligand-1
PMDs: Potentially malignant oral disorders
RISC: RNA-induced silencing complex
RNA-seq: RNA sequencing
SLNB: Sentinel lymph node biopsy
T2DM: Type 2 diabetes
TCGA: The Cancer Genome Atlas
TILs: Tumor-infiltrating lymphocytes
Tregs: T regulatory cells
TSGs: Tumor suppressor genes
UDS: Ultra-deep target sequencing
UTR: Untranslated region
WES: Whole exome sequencing
WGBS: Whole-genome bisulfite sequencing
WGS: Whole genome sequencing
